# Dynamic Tomographic Reconstruction of Deforming Volumes

**DOI:** 10.3390/ma11081395

**Published:** 2018-08-09

**Authors:** Clément Jailin, Stéphane Roux

**Affiliations:** LMT (ENS Paris-Saclay/CNRS/University Paris-Saclay), 61 avenue du Président Wilson, F-94235 Cachan, France; stephane.roux@ens-paris-saclay.fr

**Keywords:** tomographic reconstruction, dynamic tomography, motion compensation, projection-based digital volume correlation

## Abstract

The motion of a sample while being scanned in a tomograph prevents its proper volume reconstruction. In the present study, a procedure is proposed that aims at estimating both the kinematics of the sample and its standard 3D imaging from a standard acquisition protocol (no more projection than for a rigid specimen). The proposed procedure is a staggered two-step algorithm where the volume is first reconstructed using a “Dynamic Reconstruction” technique, a variant of Algebraic Reconstruction Technique (ART) compensating for a “frozen” determination of the motion, followed by a Projection-based Digital Volume Correlation (P-DVC) algorithm that estimates the space/time displacement field, with a “frozen” microstructure and shape of the sample. Additionally, this procedure is combined with a multi-scale approach that is essential for a proper separation between motion and microstructure. A proof-of-concept of the validity and performance of this approach is proposed based on two virtual examples. The studied cases involve a small number of projections, large strains, up to 25%, and noise.

## 1. Introduction

Tomography is a non-destructive imaging technique that enables the visualization of the bulk of the observed specimen. Tomography is now widely used in many fields ( e.g., medical imaging for diagnostic [1], biology [2], material science [3], etc.), performed with various waves ( e.g., X-ray, neutron, electron, terahertz, optics, ultrasound, etc.) depending on the experiment and material absorption and or scattering. Different instruments have been developed with different flux, space and time resolutions ( e.g., for X-rays medical scanners, synchrotron, lab-CT, etc.) giving access to a wide range of imaging devices and performances.

To image the 3D structure, the specimen rotates over 180° or 360° with respect the source-detector pair and at a series of distributed angles radiographs are acquired. Radiographs are transformed with dark-fields and white-fields, to extract the relative beam absorption, transformed with a logarithm (Beer-Lambert law) or more sophisticated treatments for beam hardening, in order to obtain so-called projections of the local coefficient of absorption of the sample. The collection of projections at all angles constitutes a so-called *sinogram*. Then, from the sinogram, reconstruction algorithms [4] have been developed to reconstruct the 3D imaged volume. This technique relies on the strict satisfaction of conditions, in particular concerning the geometry of the set-up and the motion of the sample as a rigid rotation with the prescribed axis and angles.

The required time for a full 3D scan varies depending on the flux (and exposition time), type of camera and rotation speed of the device. Since the beginning of the development of these techniques, the time required to acquire a tomographic scan has constantly decreased [5]. Recent papers have reported on ultra-fast tomographies, at up to 20 Hz in synchrotron beamlines, that allow extremely fast processes to be captured [6,7].

Motion of the sample during the scan is one of the main issue of tomography that leads to poor quality, blurry volumes [8]. This is the case for medical imaging (as the patient or imaged organ may move), in vivo measurements [9], for electron tomography [10] (because of the extremely small scales of observation, one cannot guarantee a fixed rotation axis at nanometer accuracy) for usually minute to hour long acquisitions, fast mechanical behavior or continuous in situ experiments [6]. Wrong or imprecise estimates of the calibration parameters (that may even vary along the scan) can also be seen as motions in the sinogram space and have the same deleterious consequences for the volume reconstruction.

Sophisticated methods have been developed to avoid or limit motion perturbations especially for periodic motion, for instance using a trigger for acquisition of radiographs based on a specific signal to captures always the same phase as can be done for cardiac or respiratory motion in medical imaging [11,12,13].

Many works have been devoted to correcting imperfect acquisitions as a post-processing treatment. For automatic (re)calibration, *online* methods, based on the motion of the sample itself during the scanning process, have been applied as a post-process after reconstruction to evaluate a corrected set of calibration parameters [14]. When dealing with electron tomography, (TEM or STEM), the voxel scale makes this problem quite limiting. The identified motion of the specimen is often regularized as being composed of rigid body motions [15,16]. However, in addition to accounting for the slight deviation of the rigid body motion of the sample from the ideal perfect rotation, motion description can be enriched by taking into account more precisely the physics of the electron trajectory in inhomogeneous magnetic fields leading to distortions [17], or sample warping due to irradiation [18,19] for electron tomography. These kinematic degrees of freedom have to be inferred at each projection, and for this fiducial markers (such as gold nanoparticles) are used. In all those cases, deformations can be treated as a slight perturbation, with strains of order of a few $10^{-3}$ at most. Similarly, in Optical Projection Tomography, OPT, Zhu et al. [20] face similar reconstruction artifacts due to motion for in vivo imaging. Motion is here regularized in time as a polynomial series, and the coefficient describing motions—essentially rigid body motions—are determined from robust quantities (geometric moments) that can be computed over the entire region of interest.

Very early, corrections were also applied in the sinogram space ([21]), with affine transforms [22]. Projection-based measurement methods (e.g., Projection-based Digital Volume Correlation (P-DVC) [23], 3D–2D registration [24,25]) have been developed to correct for rigid body motions (due to a rigid patient motion or variation of calibration parameters) from the radiograph data directly.

Yet, a deforming body with a significant strain and variation in time is a much more demanding case. Projection-based Digital Volume Correlation (P-DVC) has been shown to address part of the problem with complex 4D—3D space + time—kinematic identification [26,27,28]. First if the reference 3D geometry is well known, the displacement field can be evaluated on the fly as the sample is being deformed. This method requires a high quality reference volume and a series of deformed projections. A single projection per motion state is required to capture the full 4D (space-time) kinematics. Alternatively, imperfect acquisition conditions (but no sample strain) can also be corrected using a similar technique, without a pre-determined 3D reference geometry [23], considering that the deforming projection stack is the one used for the reconstruction.

Similar developments have been carried out very early in the context of medical imaging where periodic motion is frequent (heart beat, breathing). In particular, Refs. [29,30,31,32,33] have proposed to determine the motion of the sample from projection data. Small amplitude displacement fields with a periodic modulation in time were considered and identified using highly regularized kinematic models.

However, very often, a reference reconstructed volume is known, and is used as a prior for determining the motion [29,30,31,33,34]. This is often the case for radio-therapy treatment where the key issue is to irradiate the targeted region, in spite of a spurious motion, and hence the goal is to identify the displacement field in 3D, and a fast determination is more valuable than a very precise one.

In a similar spirit, [31,35,36] do not consider a reference to be known but rather use a phase signal (say from an electrocardiogram) to extract from a long sinogram projections coming from a similar phase of the motion, and reconstruct a low quality volume for a series of phase. Registration of the reconstructed volumes [35,36] allows the displacement field to be estimated and interpolated for the entire range of accessed phase. Then, back-correcting for this motion a deformed reconstruction grid is obtained [37] on which the projection data can be backprojected using a classical FBP/FDK algorithms [4]. In this way, each ray follows the deformed sample at each projection angle. The obtained volume has a better quality than the initial one (more details and sharper edges). Katsevich [38] proposed a mathematical study of the generalized inverse Radon transform, using a modified filtered backprojection, showing convergence in appropriate space. Further mathematical consideration lead Hahn [39] to focus on smooth boundaries of subdomains in the volume as the latters produce singularities in projections (in sinogram space, a diverging density appears along tangent planes) that can be tracked in time easily. In the following, inverse Radon transforms and filtered backprojection will not be considered, although they constitute an attractive alternative to the modified algebraic reconstruction algorithm used hereafter.

Dynamic tomography methods based on multiple volume acquisitions have been recently developed. Ruhlandt  et al. [40] proposed an approach without prior knowledge of a phase for each angle, nor of a reference volume, developed for phase contrast imaging at a synchrotron facility. The displacement field that animates a volume at time t is measured from the analysis of the motion-blurred reconstructed volume at time t−1 and t+1, then interpolated linearly. A full 4D space-time ‘movie’ of the phenomenon could be obtained. This method however requires the use of many acquired 3D volumes for the displacement field measurement, thus a high dose. The measured displacement has a relatively small amplitude compared to the volume texture characteristic scale. A criterion based on the image reconstruction quality is not easy to set and the quality has to be appreciated visually. A similar recent technique [41] deals with the correction of a volume using Digital Volume Correlation and an extended Simultaneous Algebraic Reconstruction Technique (SART) algorithm. To be able to correct a single rotation volume, the authors sub-sampled the acquired projections in 2 sub-acquisitions from which the motion is evaluated and further involved in the reconstruction strategy. This technique is however not suited to large and irregular displacements. In [42], the volume sub-sampling is performed more easily because of an especially designed sampling acquisition strategy (that cannot be adapted to any tomography). One displacement field, constant in time, is estimated for each successive pair of reconstructed volumes and is used to correct the reconstruction procedure. In this latter reference, although the tackled displacements and deformations are important during the entire test, the incremental displacement between all acquired volumes is small.

In most of these studies, the displacement and strain fields between scans was relatively small (strain of approximately 1% and uniformly distributed), and often the time (or phase) is believed to be known.

The present study proposes a strategy to reconstruct both the reference geometry and its large motion from a single sinogram. No periodic signal is used to constrain the kinematics. The recorded projections are the data that drive the measurement of the kinematic field, as is proposed in P-DVC. This however requires a “model,” here a reconstructed 3D volume, to be known in order to measure the displacement field. It is proposed here to “learn” this model from the projection data itself using a multiscale approach.

The standard reconstruction methods are briefly presented in Section 2, so that the introduction of motion can be cast in a similar framework. Section 3 details the joint determination of the reconstructed image and the motion experienced during the scan. The latter algorithm makes use of ideas comparable to those of P-DVC for the motion, and Algebraic Reconstruction Techniques (ART) for the microstructure and exploits a multiscale approach to disentangle microstructure and motion from the sinogram. Two virtual test cases of moving samples validate the procedure (Section 4). The first example is performed with the Shepp-Logan phantom with large deformation up to 20%. The second example is a checkerboard with a more complex temporal pulsating motion.

## 2. Motionless X-ray Tomography

X-ray tomographic reconstruction is based on the relative beam intensity attenuation for each discrete detector position r=[r,z] (where z is parallel to the specimen rotation axis, and r is perpendicular to it) and rotation angle. For simplicity, and because the present paper is a proof of concept, the displacement field is assumed to lie in a plane perpendicular to the rotation axis, so that each slice z remains independent from its neighbors, and the problem turns two dimensional. Hence, only one line of the detector is considered, for a unique value of z (omitted from now on).

Let us briefly recall the principle of tomography for a parallel beam: a projection p(r,θ) is defined as the line integral of f(x) along a direction $e_{\theta }$, or

(1) $$p\left(r,\theta \right)=\int_{D(r,\theta )}f\left(x\right)\;dx$$

where D(r,θ) is the line parallel to $e_{\theta }$ hitting the detector plane at position r. Different projection and interpolation algorithms exist. In the following procedure, the Matlab function radon.m is used.

Tomography consists of recording a set of $N_{\theta }$ projections p(r,θ) for a collection of angles θ(t) as the sample is rotated over a complete (or half) rotation about a fixed axis parallel to the detector plane. For a still sample, and a continuous rotation, p(r,θ(t)), written p(r,θ), is the Radon transform of f(x), p(r,θ)=R[f(x)] and hence the f(x) can be computed from an inverse Radon transform, $f\left(x\right)=R^{-1}\left[p\left(r,\theta \right)\right]$. Let us introduce the indicator function $I_{E}\left(x\right)$ of the domain E within which the volume is to be reconstructed. The ray length in E for a specific detector position r and rotation angle θ, is simply $L\left(r,\theta \right)=R_{\theta }\left[I_{E}\left(x\right)\right]$. It is useful to introduce the backprojection operator, $B_{\theta }$, which to each point x of the line D(r,θ) within E, gives a value 1/L(r,θ). Thus for any p(r,θ), $R_{\theta }\left[B_{\theta }\left[p\left(r,\theta \right)\right]\right]=p\left(r,\theta \right)$.

Tomography is now a very mature field and numerous powerful algorithms have been devised in order to deal with a discrete set of angles, with fan-bean or cone-beam projections [43], with laminography [44], etc.

However, f(x) is always assumed to stand for a rigid and still object (independent of time or rotation angle). From the collection of acquired projections, different algorithms exist to reconstruct the 3D volume [4] and fall into two categories: Fourier-domain algorithms and algebraic algorithms.

Fourier space reconstructions

With Filtered Back-Projection (FBP), each projection, p(r,θ) is first “filtered” with a ramp, or Ram-Lak filter, eventually windowed. Ignoring such windowing, in Fourier space, F[p(r,θ)](k,θ) is multiplied by |k|, inverse Fourier transformed, and then back-projected in real space, thereby producing a field $g_{\theta }\left(x\right)$ that is invariant along the direction $e_{\theta }$. These fields $g_{\theta }\left(x\right)$ are simply summed over all visited angles θ, producing the sought initial image, f(x)

(2) $$f\left(x\right)=\sum_{\theta =1}^{N_{\theta }}g_{\theta }\left(x\right)$$

Iterative reconstructions

Other reconstruction methods have received much attention, namely iterative algebraic approaches which tolerate deviations from the ideal conditions of the previous Fourier space reconstruction such as for instance having access to a continuous range of angles, covering the entire half (or full) rotation. Those methods exploit the linear structure of the problem to solve, but for computational efficiency, they avoid the writing of the linear system. They are based on the minimization over volumes, ψ(x), of the functional, $\Gamma_{\mathrm{ART}}\left[\psi \right]$, equal to the quadratic norm of the difference ρ(t,θ) between the acquired projections and the projected reconstructed volume

(3) $$\begin{array}{ccc} \Gamma_{\mathrm{ART}}\left[\psi \right] & = & \sum_{r,\theta }{∥\rho \left(t,\theta \right)∥}^{2}\\ \rho (t,\theta ) & = & p\left(r,\theta \right)-R_{\theta }\left[\psi \left(x\right)\right] \end{array}$$

then

(4) $$f=\underset{\psi }{\;\mathrm{Argmin}}\;\Gamma_{\mathrm{ART}}\left[\psi \right]$$

Additional prior information may easily be added to this functional through regularization, in order to compensate limited angle range for projections, or coarse sampling for example. This generally leads to better quality reconstructions than FBP algorithms at the expense of a higher computational cost.

To solve this huge linear inverse problem, ART algorithms essentially consist of iterative updates of the volume. Successively visiting each angle, the projection of the volume is compared with the acquired one. The difference is back projected and used to correct the volume (sometimes multiplied by a damping coefficient, not considered in our case). Faster convergence rate is observed when angles are not sampled in consecutive order but rather with a large difference between successive angles. This can be achieved for instance with a permutation of the angle order. A convergence criterion on the functional value can be used to stop the number of iterations ($\Gamma_{\mathrm{ART}}\left[f\right]<\epsilon$), with ϵ, a threshold value with respect to noise and artifact acquisition. Generally a few iterations ($N_{\mathrm{ART}}$) are required for convergence. The algorithm for this method is detailed in Algorithm 1.
**Algorithm 1:** Standard algebraic reconstruction procedure, f←ART(p).
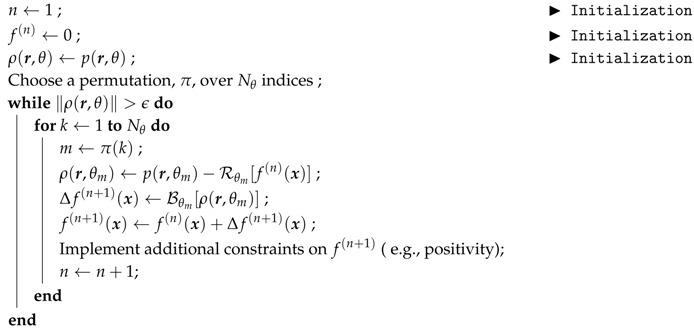


During the reconstruction procedure, additional information, defined as constraints, can be added. Those regularizations allow the reconstruction of high quality volume with a few or missing angles, noisy projections and artifacts etc. This may come from prior knowledge of the different phases of the sample (as DART algorithms proposed by [45], reconstruction with binary images [46], Total Variation [47]), dictionary learning [48], etc. However, because those regularizations are independent from the following proposed reconstruction with motion compensation, it is not considered hereafter apart from the positivity constraint f(x)←max(ψ(x),0).

## 3. Data Driven Reconstruction of Non-Rigid Samples

It is proposed to study a specimen that moves during the acquisition with a space/time displacement field u(x,t) such that, at any time, the sample is expressed with respect to a reference state f(x+u(x,t)).

For a still object f and p are bijectively related to each other through the inverse Radon transform. The introduction of motion causes a non-trivial nullspace and thus the loss of bijectivity. The reconstruction of the volume from the previously introduced algorithms ( i.e., ignoring motion) leads to a low quality, blurry, volume.

It is to be noted that the FBP reconstruction procedure has been extended to take motion into account in [35,36,40] The driving idea is to apply the back-projection step on the currently deformed geometry of the to-be-reconstructed sample, or equivalently to transport the back-projection onto the initial geometry, unwarping the motion, so that the X-ray beam would then follow non-straight paths. In Ref. [40], the motion is estimated from the registration of two reconstructions of the volume at different instants of time and linear interpolation.

(5) $$f\left(x\right)=\sum_{t=1}^{N_{\theta }}g_{\theta (t)}\left(x-u\left(x,t\right)\right)$$

Because this approach requires different volumes to estimate the displacement field, it is not suited when the motion is fast and when only a single scan can be acquired. Moreover, it is difficult to estimate a quality criterion but visual on the reconstructed volumes thus on the measured kinematics.

A recently developed Digital Volume Correlation (DVC) procedure called Projection-based DVC [26] allows to identify the 4D [49] (space-time) displacement field of sample from an initially reconstructed volume and its moving projections. An extension of this method has been applied to an *online* calibration ( i.e., calibrated from the sample motion during the scanning process) of the tomograph [23]. An initial (blurry) reconstruction was performed from a set of initial parameters. The comparison between the projection of the blurry sample and the acquired projections is, in addition to the acquisition noise and artifacts, the signature the erroneous projection geometry parameters that can be identified and corrected. The sample could be re-positioned for each angle by a rigid body motion. Because the motion was simple and of low amplitude, the correction could be applied on the sinogram itself leading to very significant improvement on the quality of the reconstruction. However, more complex displacements, or larger amplitudes (involving larger displacement variations perpendicular to the ray) would render the corrections on the projection inaccessible.

It is proposed to introduce here a new two-step algorithm based on ART reconstruction on the one hand and P-DVC on the other hand to identify both a complex and large displacement field and volume texture with a single scan performed on a moving and deforming object. The ART functional is naturally extended to account for the motion as

(6) $$\Gamma_{\mathrm{motion}-\mathrm{ART}}\left[\psi ,v\right]=\sum_{r,t}{∥R_{\theta (t)}\left[\psi \left(x+v\left(x,t\right)\right)\right]-p\left(r,t\right)∥}^{2}$$

where the summation over time extends over the $N_{t}$ acquired projections (and not just a full rotation) then

(7) $$\left(f,u\right)=\underset{\psi ,v}{\;\mathrm{Argmin}}\;\Gamma_{\mathrm{motion}-\mathrm{ART}}\left[\psi \left(x\right),v\left(x,t\right)\right]$$

The updating procedure (indexed by l) is split into two parts that are repeated alternatively:

- A volume reconstruction from an iterative *dynamic* ART algorithm assuming a known motion (described in Section 3.1);
- An update of the motion from P-DVC with a given reconstructed sample (described in Section 3.2).

However, as such, this procedure does not tolerate large displacement amplitudes. To increase the robustness and fast convergence, a multi-scale approach is coupled to the previous two-step procedure, resolving first the large scale features of both microstructure and motion, and progressively enriching the description with finer details. The complete multi-scale procedure is described in Section 3.3.

### 3.1. Dynamic Reconstruction

The dynamic reconstruction used in this article is an extension of the standard ART algorithm, and will follow the same structure as Algorithm 1. Considering the inner “for” loop, at time t (and angle θ(t), the volume is warped with the measured displacement field

(8) $${\tilde{f}}^{\left(n-1\right)}\left(x,t\right)=f^{\left(n-1\right)}\left(x+u\left(x,t\right)\right)$$

(initially u(x,t)=0). The computed projection of ${\tilde{f}}^{\left(n-1\right)}$ along θ(t) is compared with the recorded projection and the residual ( i.e., their difference)

(9) $$\rho^{\left(n\right)}\left(r,t\right)=p\left(r,t\right)-R_{\theta (t)}{\tilde{f}}^{\left(n-1\right)}\left(x,t\right)$$

is normalized and back-projected $\Delta {\tilde{f}}^{\left(n\right)}=B_{\theta (t)}\left[\rho^{\left(n\right)}\left(r,t\right)\right]$.

Finally the correction term is unwarped to the frame of the undeformed state, ${\hat{\Delta f}}^{\left(n\right)}\left(x\right)=\Delta {\tilde{f}}^{\left(n\right)}\left(x-u\right)$ so that it matches the reference configuration and it is added to the volume, $f^{\left(n\right)}=f^{\left(n-1\right)}+{\hat{\Delta f}}^{\left(n\right)}$. Let us emphasize that theoretically, ${\hat{\Delta f}}^{\left(n\right)}$ should have been defined implicitly as obeying ${\hat{\Delta f}}^{\left(n\right)}\left(x+u\right)=\Delta {\tilde{f}}^{\left(n\right)}\left(x\right)$. The two expressions are equivalent only for small strains and rotations, otherwise the unwarping should involve the Eulerian rather than the Lagrangian displacement, and one can be computed from the other. Let us also note that for not too large strains and rotations, ignoring the difference between Eulerian and Lagrangian displacements simply slows down the convergence, but the final solution is not affected. In the present case, the choice was made to use the Eulerian registration to achieve the convergence for engineering strains as large as 20%. A convergence criterion has to be chosen as in the ART procedure. Nevertheless, the criterion based on the functional value cannot be used in this case as the reconstruction is unperfect. A convergence criterion based on the variation of the functional or a maximum number of iteration $N_{\mathrm{DynART}}$ can be set. The procedure is described in Algorithm 2.
**Algorithm 2:** Proposed motion-corrected algebraic reconstruction procedure, f←DynART(p,u).
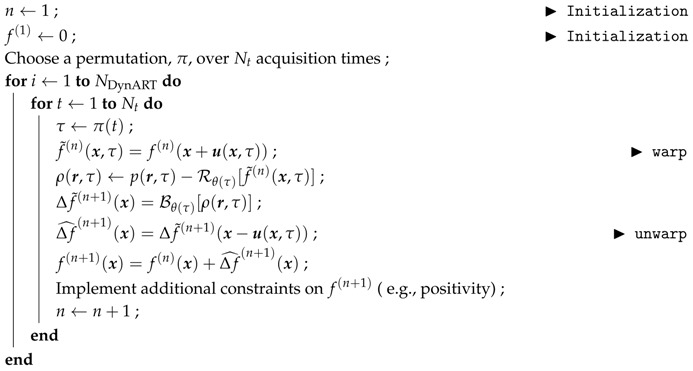


As earlier mentioned, additional priors can be added in this procedure at the end of the inner “for” loop. In the following, only a positivity constraint for f is added at each iteration.

### 3.2. Motion Identification

The full procedure is a staggered two-step process where alternatively the volume is reconstructed from a frozen displacement, and the motion is identified from a frozen estimate of the microstructure. The second step is described now.

At step l, the reconstructed volume, $f_{l}\left(x\right)$, although imperfect, is now considered as reliable. The projected residual fields $\rho_{l}\left(r,t\right)$ (computed at the end of the previous procedure when the volume is no more updated) contains patterns that are the signature of an incomplete motion correction. For the identification of the displacement field, the functional for a given f can be linearized around the previously identified displacement field $u_{l}=u_{l-1}+\delta u$

(10) $$\delta u=\underset{\delta v}{\;\mathrm{Argmin}}\sum_{r,t}{∥R_{\theta (t)}\left[\nabla {\tilde{f}}_{l}\left(x,t\right)\delta v\left(x,t\right)\right]-\rho_{l}\left(r,t\right)∥}^{2}$$

For a better conditioning, the space and time dependencies of motion may be regularized, either using “weak regularization”, with a penalty on spatial or temporal rapid variation of the displacement field to be added to the above cost function, or reverting to “strong regularization” by choosing a parametrization space composed of smooth functions of space and time. At this regularization step, any additional information pertaining to the experiment ( e.g., synchronous measurements from sensors of different modalities such as force, pressure or temperature measurements, cardiac phase etc.) can be incorporated in the kinematic model through functional dependencies on such parameters. Qualitative features may also be incorporated, for instance, the sudden occurrence of a crack, may be accounted for by allowing a temporal discontinuity in concerned degrees of freedom for the kinematics.

The chosen reduced basis is composed respectively of $N_{\tau }$ time functions, $\varphi_{i}\left(t\right)$, and $N_{s}$ vector spatial shape functions $\Phi_{j}\left(x\right)$ such as

(11) $$u\left(x,t\right)=\sum_{i=1}^{N_{\tau }}\sum_{j=1}^{N_{s}}\alpha_{ij}\varphi_{i}\left(t\right)\Phi_{j}\left(x\right)$$

with $\alpha_{ij}$ the time and space amplitudes that weight the basis functions. Setting $\varphi_{i}\left(0\right)=0$, the reference state is at initial time or angle θ=0, u(x,0)=0.

The minimization of the functional $\Gamma_{\mathrm{motion}-\mathrm{ART}}$, Equation (6), with respect to the displacement parameters δα is performed using Newton’s descent method. This procedure requires the computation of the advected image gradient and Hessian of $\Gamma_{\mathrm{motion}-\mathrm{ART}}$. They are built from the projected sensitivities

(12) $$S_{ij}\left(r,t\right)=\frac{\partial R_{\theta (t)}\tilde{f}\left(x,t\right)}{\partial \alpha_{ij}}=\varphi_{i}\left(t\right)R_{\theta (t)}\left[\Phi_{j}\left(x\right)\nabla \tilde{f}\left(x,t\right)\right]$$

Numerically, the sensitivities are computed from finite differences. The Hessian matrix and second member built from those sensitivities is

(13) $$\begin{array}{ccc} H_{ijkl} & = & \sum_{r,t}S_{ij}\left(r,t\right)S_{kl}\left(r,t\right) \end{array}$$

(14) $$\begin{array}{ccc} b_{ij} & = & \sum_{r,t}\rho \left(r,t\right)S_{ij}\left(r,t\right) \end{array}$$

thus the vector of displacement amplitude correction δα is the solution of the linear system

(15) [H]δα=b

from which the displacement is updated. This procedure is repeated until the projection residual is no longer decreasing. Algorithm 3 summarizes the determination of the displacement field. In the following test cases, a single iteration in this algorithm is performed before updating the volume.
**Algorithm 3:** Displacement identification procedure, $u\leftarrow \mathrm{PDVC}(f_{\lambda },p,u_{0})$.
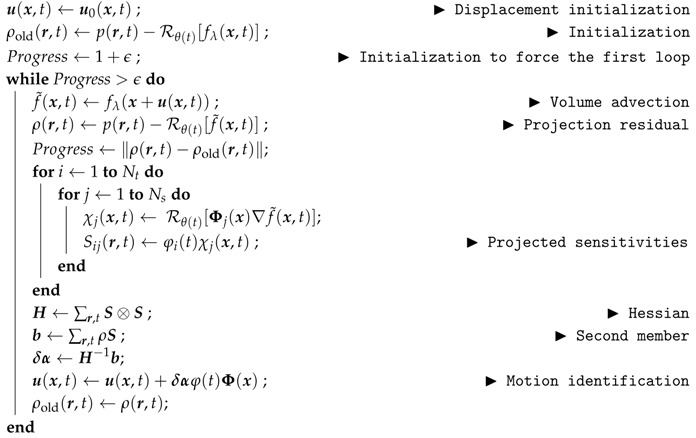


### 3.3. Multi-Scale Approach

If displacement magnitude is bounded by a length scale λ, one expects that the reconstruction is fair at a scale larger than λ. Hence, if the original image is convoluted with a Gaussian of width λ, it should well match its sinogram. One convenient property of the projection is that the projection of the convoluted image is the convolution of the original projection with a Gaussian of the same width. However, because of motion, this matching is not perfect but just fair. It means that one may estimate a better match by treating the deformation as a *slight* perturbation.

More precisely, the recorded projections are convoluted by the Gaussian of width λ,

(16) $${\overset{˘}{p}}_{\lambda }\left(r,\theta \right)=\sum_{r^{\prime }}G_{\lambda }\left(r^{\prime }\right)p\left(r-r^{\prime },\theta \right)$$

where, $G_{\lambda }\left(r\right)=1/\left(2\pi \lambda^{2}\right){\exp(-|r|}^{2}/\left(2\lambda^{2}\right))$.

Using the progressively identified displacement field, a more accurate determination of f can be achieved using the above described reconstruction. Because a large part of the displacement is expected to be captured in u, the idea is to repeat the above procedure but with a smaller Gaussian filter, namely cutting down λ by a factor of two. Thus at each iteration, the displacement correction being smaller and smaller, convergence to the actual displacement field is expected. A convergence criterion is chosen on the norm of the residual variation or on the norm of the displacement correction.

The summary of the complete procedure is described in Algorithm 4.
**Algorithm 4:** Complete dynamic tomography procedure, (f,u)←DynTomo(p).
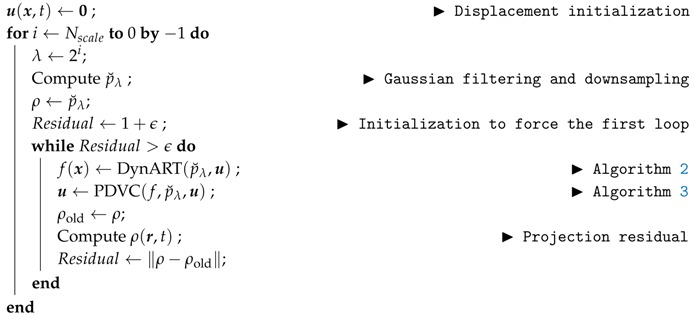


## 4. Test Case

Two numerical test cases are proposed to validate the procedure. To build the input data, two geometries are chosen, and two kinematics (one per case) deformed and projected at all considered angle. The obtained projections are then corrupted by a white Gaussian noise (standard deviation of 1% of the gray level dynamic of the projections and are used as the virtual experimental inputs for our procedure.

Both examples are carried out on 512×512 pixel images. The beam is parallel, and only $N_{\theta }=300$ projections are acquired over a single 360° rotation.

- The first application corresponds to a moving Shepp-Logan phantom with large displacement magnitude (up to 37 pixels) and large engineering strains (27%). Large strains are chosen here in order to highlight the robustness of the proposed procedure as compared with previously studied examples where strains were about 1% [40].
- The second test is performed on a checkerboard with smaller displacements but a more complex time evolution composed of two separated modes: a steady drift superimposed to a high frequency pulsating motion.

In both test cases, the displacement bases chosen for the inverse problem were similar to the ones used for performing the direct problem, so that no additional model error (apart from noise) is introduced. The space functions Φ(x) are composed of four C4 mesh elements (4-node square elements with bilinear interpolations). The space basis $N_{s}$ is hence composed of 18 degrees of freedom.

### 4.1. Shepp-Logan Phantom Case

In this test case, the Shepp-Logan phantom is used and deformed up to 27%. For this test case, a single time evolution (linear drift in time) is applied. The imposed displacement field can be written

(17) $$v\left(x,\theta \right)=\theta /N_{\theta }\cdot v\left(x\right)$$

The nine nodal displacements are given in Table 1 in x and y directions.

**Figure 1:**
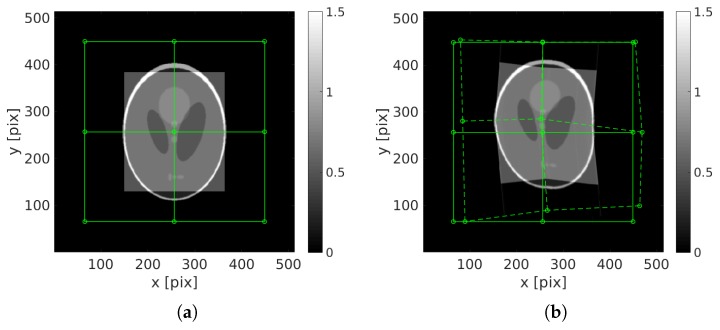
(**a**) Reference image and the 9-node mesh the node of which are subjected to a random displacement, assumed to be linear in time; (**b**) deformed phantom at final time $N_{\theta }$.

**Figure 2:**
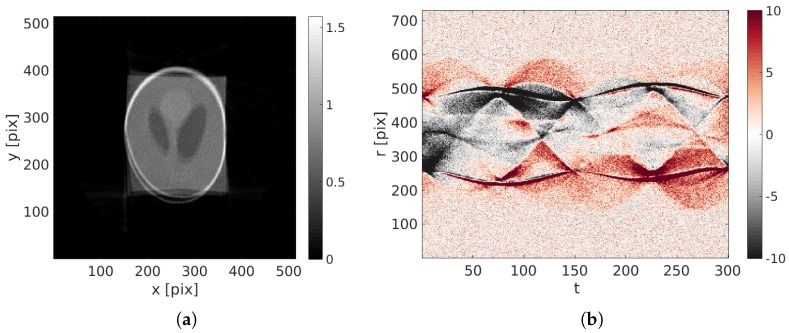
(**a**) Initial reconstruction with u(x,t)=0; (**b**) initial projected residual fields ρ(r,t). Please note that the color amplitude that is saturated in this image has been selected to be the same with the corrected residuals shown further down (Figure 3).

The reference and deformed phantoms are shown Figure 1. The maximum displacement amplitude is 37 pixels. The first reconstruction of the image (standard ART procedure), presented Figure 2a is very blurry. Some parts of the phantom are split in two. The initial projected residual fields are very high and stresses that the reconstruction is not properly performed.

**Figure 3:**
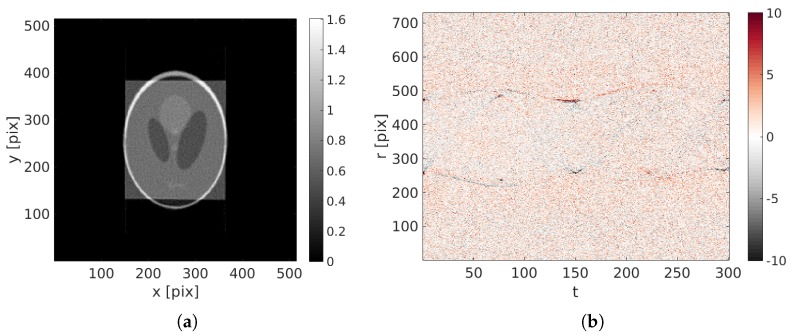
(**a**) Reconstructed image with the identified displacement field; (**b**) final projected residual fields ρ(r,t).

Before using the proposed procedure, the multi-scale procedure presented Section 3.3 is applied to the projections to willingly blur the reconstruction. After 60 iterations ( i.e., volume updates), the displacement field has converged. The corrected reconstructed volume is presented Figure 3. The edges are sharp and the gray level amplitudes are correct. The projected residual fields (true metric of our procedure) is mostly composed of the white Gaussian noise meaning that the proposed procedure has been successful.

The displacement error computed on the nodal values displays a standard deviation of 3.10 pixel. This result validates the procedure.

**Figure 4:**
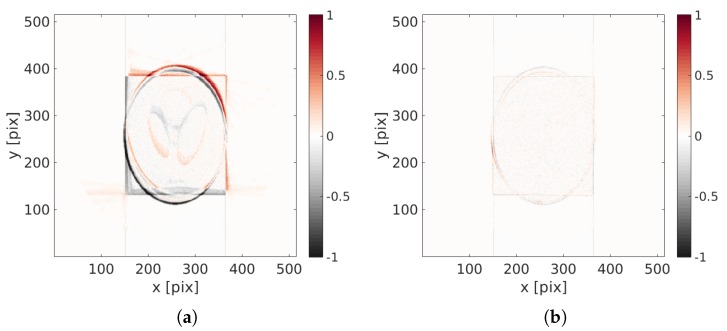
Difference between the reference volume and the (**a**) initial ( i.e., ART(p)) and (**b**) final ones (i.e., DynTomo(p)).

As a last validation of the phantom reconstruction quality, the reconstruction is compared to the reference volume f. It is shown in Figure 4 that the reconstructed shape and positioning is very good. The final difference displays a “ghost” of the phantom that points out a small intensity error that does not appear in the residual fields.

### 4.2. Pulsating Checkerboard Case

This second test case is here based on a checkerboard composed of 8×8 squares of 35×35 pixels each. This square shaped pattern is chosen to exhibit reconstruction errors very clearly since sharp and straight boundaries are very easily detected, and hence the visual perception is a very severe test.

In this example, the imposed (supposed unknown) displacement field is composed of the sum of two parts:

- A pulsating motion: Temporally, a shifted cosine function (1−cos…)) (obeying the constraint of being null at time 0) evolution with a non-integer number of periods to avoid symmetry (here 2.35 periods during the full-rotation scan). Spatially, the displacement field is a centered dilatation/contraction;
- A linear drift in time for all nodes with random directions and amplitudes.

The applied displacement field can be written

(18) $$u\left(x,\theta \right)=\left(1-\cos(2.35\cdot 2\pi \cdot \theta /N_{\theta })\right)\cdot V_{1}\left(x\right)+\theta /N_{\theta }\cdot V_{2}\left(x\right)$$

with the nodal values presented in Table 2 and Table 3.

**Figure 5:**
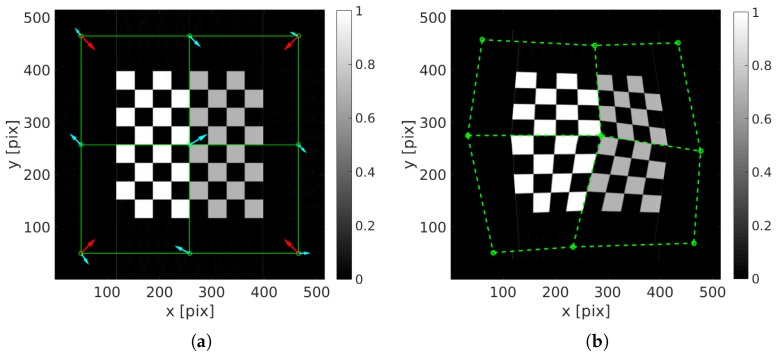
(**a**) Reference image (unknown) and applied nodal displacement field $V_{1}\left(x\right)$ in red and $V_{2}\left(x\right)$ in light blue; (**b**) deformed checkerboard at time $N_{t}$.

The nodal displacement vectors $V_{1}\left(x\right)$ and $V_{2}\left(x\right)$ are shown in Figure 5a. The reference image, the deformed one at the end of the scan and the chosen C4 mesh are shown in Figure 5. The maximum strain is about 25%.

**Figure 6:**
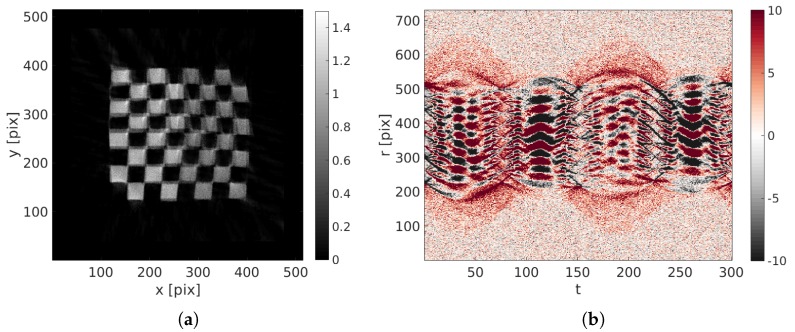
(**a**) Initial reconstruction with u(x,t)=0; (**b**) initial projected residual fields ρ(r,t).

Because of the large motion amplitude, the initial reconstruction ( i.e., obtained from a standard ART procedure for which u(x,θ)=0) is fuzzy and its quality is very poor as can be judged from Figure 6a. The projection of this blurred volume is compared with the initial projection to generate the initial projected residual fields ρ(r,t) (see Figure 6b).

After 60 iterations, ( i.e., 60 updates of the reconstruction) — performed in approximately 2 h—the 38 degrees of freedom that drive the displacement field (18 spatial times 2 temporal degrees of freedom) have converged to a steady value. A small standard deviation of the displacement field error with respect to the prescribed displacement of less than 1.2 pixel remains at the end. Considering the large imposed motion amplitude, the estimated kinematics is deemed quite satisfactory.

**Figure 7:**
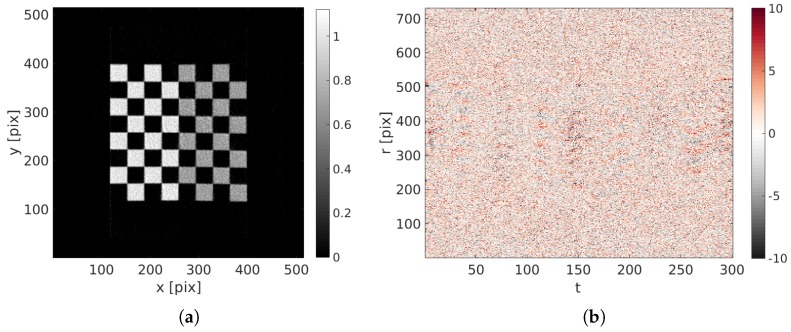
(**a**) Reconstructed image with the identified displacement field; (**b**) final projected residual fields ρ(r,t).

**Figure 8:**
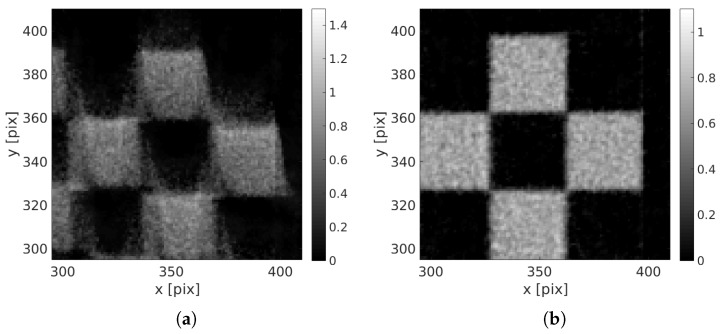
Zoom in the reconstructed volume (**a**) with a standard non-corrected volume and (**b**) with the proposed procedure.

The final reconstruction and projected residuals are shown in Figure 7. The reconstruction has sharp edges and its constituting squares have been correctly reconstructed. Zooms in the initial and corrected specimen are shown in Figure 8. The projected residual field, where all features of the initial sinogram have been completely erased, and only white Gaussian noise remains, means that the reconstruction has been quite successful.

**Figure 9:**
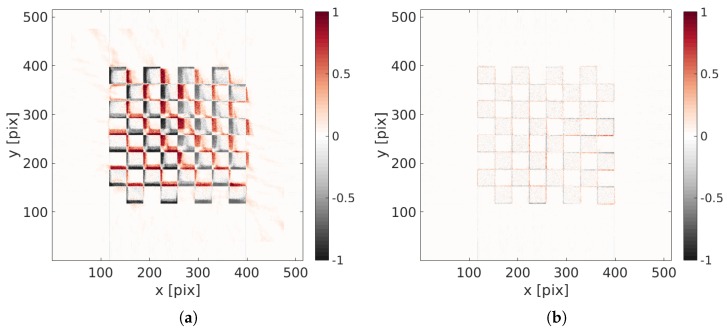
Difference between the initial and perfect image and (**a**) the initial reconstruction (i.e., ART(p)) and (**b**) the achieved volume (i.e., DynTomo(p)). A good positioning is reached at the end.

To correctly appreciate the quality of the achieved volume, a difference with the initial perfect one is shown Figure 9. This difference highlights a perfect positioning of the reconstruction, and only slight discrepancies of the gray level intensity on the bright squares are visible.

The full procedure (i.e., 60 complete iterations for 512 × 512 pixel images) is performed in approximately 1 h. What takes time (and iterations) is the computation of the sensitivities that requires the deformed volumes over the entire range of time. However, it is worth mentioning that the code has not been optimized, since only a proof of concept was aimed at.

## 5. Discussion and Conclusions

An innovative algorithm is presented to perform simultaneously a dynamic reconstruction of a moving sample with the identification of the full 2D space and time displacement field. The method is derived from Algebraic Reconstruction Techniques coupled with Projection based Digital Volume Correlation. The iterative algorithm is based on two steps:

- For a given displacement field, a dynamic algebraic reconstruction algorithm is proposed. Each iteration of the procedure consists in comparing the acquired projection with the projected warped volume (deformed with current displacement field). The projected residual is backprojected, unwarped to match the reference space and added to the volume;
- For a given reference volume, a P-DVC analysis allows the displacement field to be identified. The projection of the (unperfect) warped volume is compared with the acquired projections. The residual can be read as motion using the computed sensitivity fields. An update of the displacement field is then performed.

A multiscale procedure has been proposed as an essential ingredient to properly correct large displacements. The acquired projections are first convoluted with a Gaussian kernel of large width (low pass filter) to increase its correlation length and capture large corrections from the linearized P-DVC functional. The Gaussian filter is then progressively reduced, following the residual norm evolution, to identify finer details.

The post treatment procedure, that exploits the same data as a standard acquisition (same number of projections and standard projection operator), has been tested with two challenging numerical examples (with large displacements and strains). The first is a Shepp Logan phantom with large displacement fields (up to 1/4 of the phantom length). The second is a checkerboard with a pulsating motion in time. Both examples are corrupted by a white Gaussian noise that probes the robustness with respect to the acquisition noise. The two applications show a nearly perfect identification of the displacement field and dynamic reconstruction. Performed with a parallel projection algorithm for simplicity, the exact same method can be applied with any projection model.

The proposed dynamic reconstruction algorithm has been devised as an extension of the ART algorithms. It is convenient with those approaches to include in the process an *a priori* knowledge of the scanned specimen (assumption on the gray levels, its variations, the number of phases, its sizes, etc.). Many different regularization have been proposed in the literature that enable to obtain high quality reconstructions, with less artifacts, from less projections or missing angles, etc. Because those regularizations are independent of the current algorithms, it was chosen not to implement them and focus on the proposed method performances without any ’additional help’. Nevertheless, they are fully compatible with the proposed approach and can be implemented in a transparent fashion. When aiming to perform ultra-fast acquisitions with a few angles, they would certainly be very precious to accelerate convergence, and improve reconstruction quality.

In the proposed examples, the optical flow was kept constant. Some applications may require to include a gray level variation model. A perspective of this work could be the scan of in situ mechanical test with high strains, the identified deformation could be used to correct for absorption evolution of the material considering a constant beam intensity.

The proposed procedure shows performances that can be beneficial to numerous fields. The clear reconstruction of the moving sample allows for qualitative and quantitative analyses:

- Combined with Digital Volume Correlation [50] between well reconstructed volumes;
- Combined with image segmentation for diagnosis from radiology;
- Combined with ultra-fast tomography acquisition as recently available from some synchrotron beam-lines [6,51].

This is key for data assimilation [52] and model identification and validation in material science [53] with CT-scan as already developed with MRI [54].

## Figures and Tables

**Table 1:** Applied nodal displacements for V(x) in pixels, in x (left) and y (right).

*y\x*	**66**	**256**	**446**		*y\x*	**66**	**256**	**446**
**446**	15	0	4		**446**	4	0	1
**256**	19	−4	19		**256**	26	30	0
**66**	26	10	15		**66**	0	24	34

**Table 2:** Applied nodal displacements for $V_{1}\left(x\right)$ in pixels, in x (left) and y (right).

*y\x*	50	256	462		*y\x*	50	256	462
**462**	28	0	−28		**462**	−28	0	−28
**256**	0	0	0		**256**	0	0	0
**50**	28	0	−28		**50**	28	0	28

**Table 3:** Applied nodal displacements for $V_{2}\left(x\right)$ in pixels, in x (left) and y (right).

*y\x*	50	256	462		*y\x*	50	256	462
**462**	-13	22	−17		**462**	17	−22	9
**256**	−22	34	17		**256**	22	22	−17
**50**	17	−30	22		**50**	−22	17	0
